# Crystal structures of Na_2_SeO_4_·1.5H_2_O and Na_2_SeO_4_·10H_2_O

**DOI:** 10.1107/S1600536814011799

**Published:** 2014-07-19

**Authors:** Matthias Weil, Barbara Bonneau

**Affiliations:** a Institute for Chemical Technologies and Analytics, Division of Structural Chemistry, Vienna University of Technology, Getreidemarkt 9/164-SC, A-1060 Vienna, Austria; b IUT Bordeaux 1, 15 Rue Naudet, 33175 Gradignan, France

**Keywords:** isotypism, sodium selenate, salt hydrates, crystal structure

## Abstract

The crystal structures of Na_2_SeO_4_·1.5H_2_O (sodium selenate sesquihydrate) and Na_2_SeO_4_·10H_2_O (sodium selenate deca­hydrate) are isotypic with those of Na_2_CrO_4_·1.5H_2_O and Na_2_ *X*SeO_4_·10H_2_O (*X* = S, Cr), respectively. The asymmetric unit of the sesquihydrate contains two Na^+^ cations, one SeO_4_ tetra­hedron and one and a half water mol­ecules, the other half being generated by twofold rotation symmetry. The coordination polyhedra of the cations are a distorted monocapped octa­hedron and a square pyramid; these [NaO_*x*_] polyhedra are linked through common edges and corners into a three-dimensional framework structure, the voids of which are filled with the Se atoms of the SeO_4_ tetra­hedra. The structure is consolidated by O—H⋯O hydrogen bonds between coordinating water mol­ecules and framework O atoms. The asymmetric unit of the deca­hydrate consists of two Na^+^ cations, one SeO_4_ tetra­hedron and ten water mol­ecules. Both Na^+^ cations are octa­hedrally surrounded by water mol­ecules and by edge-sharing condensed into zigzag chains extending parallel to [001]. The SeO_4_ tetra­hedra and two uncoordinating water mol­ecules are situated between the chains and are connected to the chains through an intricate network of medium-strength O—H⋯O hydrogen bonds.

## Abstract 2

The crystal structures of the 1.5- and 10-hydrates of Na_2_SeO_4_ are isotypic with those of the corresponding chromates.

## Chemical context   

Based on recent studies in the system Na/Se/O/H that revealed dimorphism of the phases NaHSeO_4_ and Na_5_H_3_(SeO_4_)_4_(H_2_O)_2_ (Pollitt & Weil, 2014 ▶), we became inter­ested in the structure determination of hydrous phases of Na_2_SeO_4_. Although the first report of the deca­hydrate of Na_2_SeO_4_ dates back to 1827 (Mitscherlich, 1827 ▶), a detailed structure report for this compound has not been published so far. Mitscherlich (1827 ▶) also recognized an isomorphic relationship of Na_2_SeO_4_·10H_2_O with Na_2_SO_4_·10H_2_O (Glauber’s salt or mirabilite as a mineral species). This relation was later confirmed by Rosický (1908 ▶) and by Ruben *et al.* (1961 ▶) on the basis of unit-cell determinations using diffraction methods. Another hydrous phase of Na_2_SeO_4_ reported in the literature is the metastable hepta­hydrate that crystallized from supersaturated Na_2_SeO_4_ solutions only when seeded with Na_2_SO_4_·7H_2_O nuclei below 293 K (Belarew, 1965 ▶).

During crystallization studies of aqueous Na_2_SeO_4_ solutions under different temperature conditions, we were able to isolate crystals not only of the deca­hydrate, but also of the sesqui­hydrate, the crystal structures of which are reported here.

## Structural commentary   

**Figure 1:**
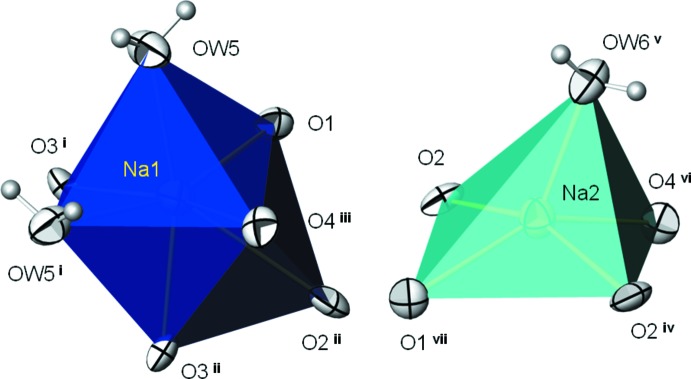
The NaO_7_ and NaO_5_ polyhedra in the structure of Na_2_SO_4_·1.5H_2_O. Displacement parameters are drawn at the 99% probability level. [Symmetry codes: (i) 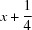, 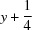, 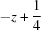; (ii) 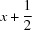, 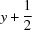, *z*; (iii) 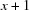, *y*, *z*; (iv) 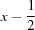, 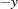, 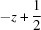; (v) 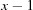, *y*, *z*; (vi) *x*, 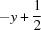, 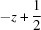; (vii) 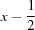, 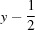, *z*.]

**Figure 2:**
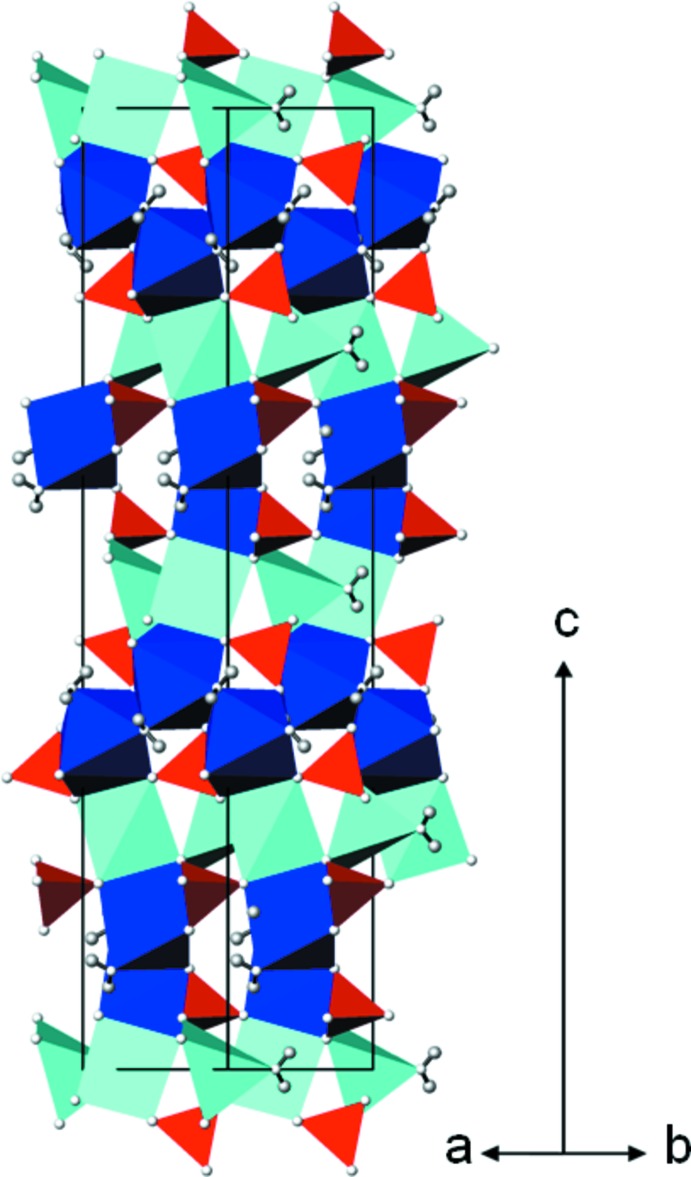
The crystal structure of Na_2_SO_4_·1.5H_2_O in a projection along [110]. NaO_5_ polyhedra are turquoise, NaO_7_ polyhedra are blue, SeO_4_ tetra­hedra are red and H atoms are grey. Hydrogen bonds have been omitted for clarity.

Na_2_SeO_4_·1.5H_2_O is isotypic with the corresponding chromate (Kahlenberg, 2012 ▶) and is the second example of the Na_2_ *X*O_4_·1.5H_2_O structure family. The main building blocks of this structure type are distorted [NaO_5_(H_2_O)_2_] (Na1) monocapped octa­hedra, distorted [NaO_4_(H_2_O)] square pyramids (Na2) (Fig. 1 ▶) and rather regular *X*O_4_ (*X* = Se, Cr) tetra­hedra. These building blocks are linked through common corners and edges into a three-dimensional framework structure (Fig. 2 ▶). Hydrogen bonds of the type O—H⋯O between the coord­in­ating water mol­ecules and parts of the framework O atoms provide additional stabilization (Table 1 ▶). The bond lengths (Table 2 ▶) and angles within the individual building blocks of the selenate and chromate structures are more or less identical with mean distances of SeO_4_ = 1.641; CrO_4_ = 1.651; Na1O_7_ = 2.514 (selenate), 2.505 (chromate); Na2O_5_ = 2.350 (selenate), 2.360 Å (chromate).

**Figure 3:**
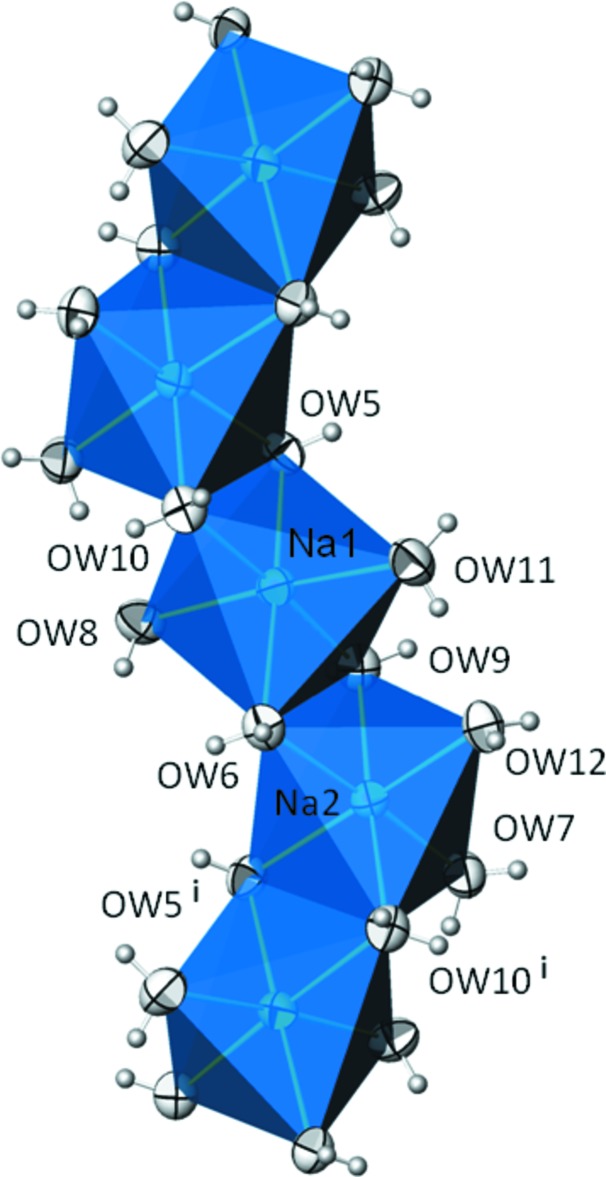
A chain of edge-sharing NaO_6_ octa­hedra in the crystal structure of Na_2_SO_4_·10H_2_O. Displacement parameters are drawn at the 99% probability level. [Symmetry code: (i) *x*, −*y* − 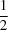, *z* − 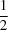.]

**Figure 4:**
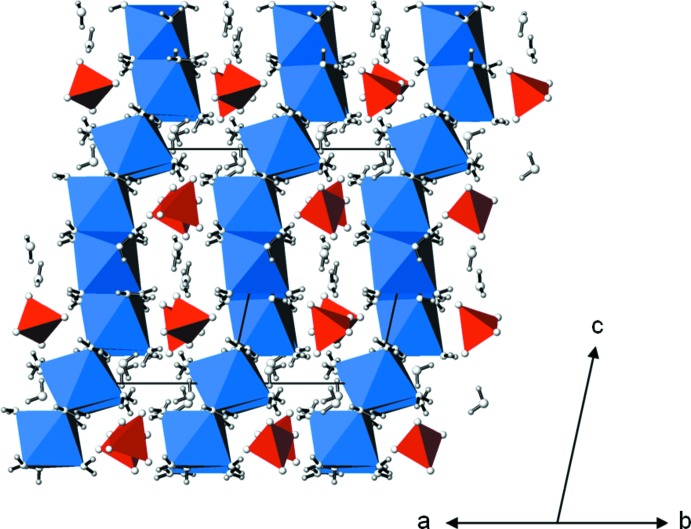
The crystal structure of Na_2_SO_4_·10H_2_O in a projection along [110]. NaO_6_ polyhedra are light blue, SeO_4_ tetra­hedra are red, O atoms are white and H atoms are grey. Hydrogen bonds have been omitted for clarity.

Isotypism has been reported for several Na_2_ *X*O_4_·10H_2_O (*X* = S, Se, Cr, W, Mo) phases (Ruben *et al.*, 1961 ▶), but only the structures of *X* = S (Levy & Lisensky, 1978 ▶; Prescott *et al.*, 2001 ▶) and Cr (Kahlenberg, 2012 ▶) have been determined so far. As expected, the general structural set-up in the isotypic Na_2_ *X*O_4_·10H_2_O structures is very similar. Each of the two Na^+^ cations is octa­hedrally surrounded [mean Na—O distance of the two octa­hedra is 2.420 Å (see Table 3 ▶); sulfate analogue (Prescott *et al.*, 2001 ▶): 2.415 Å; chromate analogue (Kahlenberg, 2012 ▶): 2.423 Å]. The [NaO_6_] octa­hedra are linked *via* edge-sharing into zigzag chains (Fig. 3 ▶) running parallel to [001]. These chains are linked with neighbouring chains and inter­mediate SeO_4_ tetra­hedra (mean Se—O distance 1.639; sulfate 1.488, chromate 1.647 Å) and non-coordinating lattice water mol­ecules through O—H⋯O hydrogen bonds of medium strength (Table 4 ▶) to build up the crystal structure (Fig. 4 ▶). The most important difference between the structures of the three Na_2_ *X*O_4_·10H_2_O (*X* = S, Se, Cr) phases is the missing disorder of the *X*O_4_ tetra­hedron in the selenate compound that has been observed in the sulfate compound on the basis of single-crystal neutron data (Levy & Lisensky, 1978 ▶) and single-crystal X-ray data (Prescott *et al.*, 2001 ▶), or for the chromate compound on the basis of single-crystal X-ray data (Kahlenberg, 2012 ▶).

## Synthesis and crystallization   

Anhydrous Na_2_SeO_4_ was prepared according to the method compiled by Brauer (1963 ▶) by adding a half-concentrated aqueous selenic acid solution (*ca* 60 wt%) to an excess of an Na_2_CO_3_ solution. The resulting solution was heated until a considerable amount of the neutralization product had crystallized. The crystal mush was then separated by suction filtration of the still-hot solution and dried in air. X-ray powder diffraction revealed a single-phase material. The Na_2_SeO_4_ crystals were then dissolved in small amounts of water and kept at *ca* 300, 293 and 280 K until complete evaporation of the solvent. According to Rietveld refinements using *TOPAS* (Bruker, 2013 ▶) the product crystallized at 300 K consisted of Na_2_SeO_4_ and Na_2_SeO_4_·1.5H_2_O in an approximate 9:1 weight ratio, the product crystallized at 290 K consisted of Na_2_SeO_4_ and Na_2_SeO_4_·1.5H_2_O in an approximate 5:1 ratio, and the product crystallized at 280 K consisted of Na_2_SeO_4_, Na_2_SeO_4_·1.5H_2_O and Na_2_SeO_4_·10H_2_O in an approximate 5:4:1 ratio. The crystal forms of the three obtained phases were different and were used for separation. Crystals of the anhydrous phase had mainly a lath-like form, of the sesquihydrate a plate-like form, and of the deca­hydrate a pinacoidal form. All obtained hydrate phases tend to weather when stored under ambient conditions.

## Refinement   

Unit-cell determinations revealed isotypic relationships with the corresponding chromate phases (Kahlenberg, 2012 ▶). For better comparison of the isotypic structures, atom labels and the setting of the unit cells of the selenate compounds were retained, and the coordinates of the non-H atoms of the chromate structure were used as starting parameters for refinement [note that the unit cell of Na_2_CrO_4_·1.5H_2_O is given in the non-standard setting *F*2*dd* of space group No. 43 (standard setting *Fdd*2)]. The H atoms of the water mol­ecules were located from difference maps and were refined with a common *U*_iso_ parameter and a fixed O—H distance of 0.82 Å. Experimental details are given in Table 1 ▶.

## Supplementary Material

Crystal structure: contains datablock(s) 1.5-hydrate, 10-hydrate, global. DOI: 10.1107/S1600536814011799/hb0010sup1.cif

Structure factors: contains datablock(s) 1.5-hydrate. DOI: 10.1107/S1600536814011799/hb00101.5-hydratesup2.hkl

CCDC references: 1004274, 1004275

Additional supporting information:  crystallographic information; 3D view; checkCIF report

## Figures and Tables

**Table 1:** Hydrogen-bond geometry (Å, °) for 1.5-hydrate

*D*—H⋯*A*	*D*—H	H⋯*A*	*D*⋯*A*	*D*—H⋯*A*
O*W*5—H1⋯O4^viii^	0.82 (1)	2.13 (1)	2.922 (2)	164 (3)
O*W*5—H2⋯O3^ix^	0.82 (1)	2.08 (1)	2.891 (2)	169 (3)
O*W*6—H3⋯O1^vi^	0.82 (1)	1.90 (1)	2.703 (2)	167 (4)

Symmetry codes: (vi) 
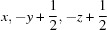
; (viii) 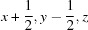; (ix) 
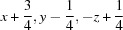
.

**Table 2:** Selected bond lengths (Å) for 1.5-hydrate

Na1—O*W*5	2.3660 (18)	Na2—O2^iv^	2.3301 (18)
Na1—O3^i^	2.4157 (19)	Na2—O*W*6^v^	2.3480 (18)
Na1—O1	2.4379 (18)	Na2—O4^vi^	2.3651 (19)
Na1—O3^ii^	2.4594 (16)	Na2—O1^vii^	2.4103 (18)
Na1—O*W*5^i^	2.465 (2)	Se1—O2	1.6350 (14)
Na1—O4^iii^	2.6057 (19)	Se1—O3	1.6367 (14)
Na1—O2^ii^	2.8475 (17)	Se1—O4	1.6451 (16)
Na2—O2	2.298 (2)	Se1—O1	1.6481 (15)

Symmetry codes: (i) 
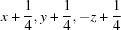
; (ii) 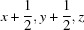; (iii) 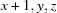; (iv) 
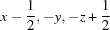
; (v) 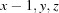; (vi) 
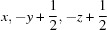
; (vii) 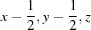.

**Table 3:** Selected bond lengths (Å) for 10-hydrate

Na1—O*W*5	2.3776 (6)	Na2—O*W*7	2.3935 (6)
Na1—O*W*6^i^	2.4181 (6)	Na2—O*W*9	2.4325 (6)
Na1—O*W*11	2.4184 (6)	Na2—O*W*6	2.4415 (6)
Na1—O*W*10	2.4194 (6)	Na2—O*W*10^ii^	2.4667 (6)
Na1—O*W*8^i^	2.4473 (6)	Se1—O41	1.6335 (5)
Na1—O*W*9^i^	2.4507 (6)	Se1—O31	1.6394 (5)
Na2—O*W*12	2.3814 (6)	Se1—O1	1.6398 (5)
Na2—O*W*5^ii^	2.3891 (6)	Se1—O21	1.6421 (5)

Symmetry codes: (i) 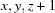; (ii) 
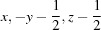
.

**Table 4:** Hydrogen-bond geometry (Å, °) for 10-hydrate

*D*—H⋯*A*	*D*—H	H⋯*A*	*D*⋯*A*	*D*—H⋯*A*
O*W*5—H5*A*⋯O41	0.82 (1)	1.96 (1)	2.7570 (7)	164 (1)
O*W*5—H5*B*⋯O*W*13^iii^	0.82 (1)	2.00 (1)	2.7980 (7)	165 (1)
O*W*6—H6*A*⋯O*W*14	0.82 (1)	2.02 (1)	2.8301 (7)	168 (1)
O*W*6—H6*B*⋯O41^ii^	0.82 (1)	1.98 (1)	2.7791 (7)	166 (2)
O*W*7—H7*A*⋯O1^iv^	0.82 (1)	1.97 (1)	2.7727 (7)	166 (1)
O*W*7—H7*B*⋯O*W*8^v^	0.82 (1)	1.95 (1)	2.7542 (7)	168 (1)
O*W*8—H8*A*⋯O41^ii^	0.82 (1)	1.95 (1)	2.7544 (7)	166 (1)
O*W*8—H8*B*⋯O*W*7^vi^	0.82 (1)	1.99 (1)	2.8076 (7)	178 (1)
O*W*9—H9*A*⋯O1^vii^	0.82 (1)	2.11 (1)	2.9152 (7)	168 (1)
O*W*9—H9*B*⋯O*W*13^viii^	0.82 (1)	2.04 (1)	2.8596 (7)	177 (1)
O*W*10—H10*A*⋯O*W*14^ix^	0.82 (1)	2.05 (1)	2.8686 (7)	178 (2)
O*W*10—H10*B*⋯O31^x^	0.82 (1)	2.08 (1)	2.8920 (7)	174 (1)
O*W*11—H11*A*⋯O31	0.82 (1)	2.05 (1)	2.8604 (7)	171 (1)
O*W*11—H11*B*⋯O*W*12^i^	0.82 (1)	1.96 (1)	2.7716 (8)	168 (1)
O*W*12—H12*A*⋯O21^iv^	0.82 (1)	1.92 (1)	2.7359 (7)	179 (1)
O*W*12—H12*B*⋯O*W*11^viii^	0.82 (1)	1.97 (1)	2.7818 (7)	173 (1)
O*W*13—H13*A*⋯O1^xi^	0.82 (1)	1.98 (1)	2.7932 (7)	172 (1)
O*W*13—H13*B*⋯O21^x^	0.82 (1)	1.98 (1)	2.7931 (7)	170 (1)
O*W*14—H14*A*⋯O21^xii^	0.82 (1)	1.98 (1)	2.8002 (7)	174 (1)
O*W*14—H14*B*⋯O31^viii^	0.82 (1)	2.00 (1)	2.8061 (7)	169 (1)

Symmetry codes: (i) 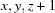; (ii) 
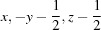
; (iii) 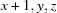; (iv) 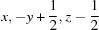; (v) 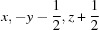; (vi) 
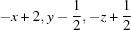
; (vii) 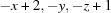; (viii) 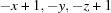; (ix) 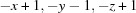; (x) 
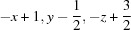
; (xi) 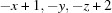; (xii) 
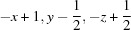
.

**Table 5:** Experimental details

	1.5-hydrate	10-hydrate
Crystal data		
Chemical formula	Na_2_SeO_4_·1.5H_2_O	Na_2_O_4_Se·10H_2_O
*M*_r_	215.96	369.10
Crystal system, space group	Orthorhombic, *F*2*d* *d*	Monoclinic, *P*2_1_/*c*
Temperature (K)	100	100
*a*, *b*, *c* (Å)	6.7533 (8), 8.6299 (10), 35.206 (4)	11.5758 (6), 10.4911 (5), 12.9570 (7)
α, β, γ (°)	90, 90, 90	90, 107.995 (3), 90
*V* (Å^3^)	2051.8 (4)	1496.56 (13)
*Z*	16	4
Radiation type	Mo *K*α	Mo *K*α
μ (mm^−1^)	7.43	2.62
Crystal size (mm)	0.20 × 0.15 × 0.10	0.32 × 0.18 × 0.09

Data collection		
Diffractometer	Bruker SMART CCD	Bruker APEXII CCD
Absorption correction	Multi-scan (*SADABS*; Bruker, 2008 ▶)	Multi-scan (*SADABS*; Bruker, 2013 ▶)
*T*_min_, *T*_max_	0.488, 0.584	0.642, 0.749
No. of measured, independent and observed [*I* > 2σ(*I*)] reflections	8363, 1824, 1723	213856, 11218, 9196
*R*_int_	0.032	0.054
(sin θ/λ)_max_ (Å^−1^)	0.762	0.965

Refinement		
*R*[*F* ^2^ > 2σ(*F* ^2^)], *wR*(*F* ^2^), *S*	0.019, 0.042, 0.99	0.021, 0.046, 1.05
No. of reflections	1824	11218
No. of parameters	89	215
No. of restraints	4	20
H-atom treatment	H-atom parameters constrained	H-atom parameters constrained
Δρ_max_, Δρ_min_ (e Å^−3^)	0.91, −0.37	0.48, −0.52
Absolute structure	Flack (1983 ▶), 823 Friedel pairs	–
Absolute structure parameter	0.025 (8)	–

Computer programs: *SMART*, *SAINT*, *SAINT-Plus* and *APEX2* (Bruker, 2013 ▶, 2013 ▶), *SHELXS97* and *SHELXL97* (Sheldrick, 2008 ▶), *ATOMS* (Dowty, 2006 ▶) and *publCIF* (Westrip, 2010 ▶).

## supplementary crystallographic information

### Crystal data

**Table:**

Na_2_O_4_Se·10H_2_O	*F*(000) = 752
*M**_r_* = 369.10	*D*_x_ = 1.638 Mg m^−^^3^
Monoclinic, *P*2_1_/*c*	Mo *K*α radiation, λ = 0.71073 Å
Hall symbol: -P 2ybc	Cell parameters from 9719 reflections
*a* = 11.5758 (6) Å	θ = 2.7–40.1°
*b* = 10.4911 (5) Å	µ = 2.62 mm^−^^1^
*c* = 12.9570 (7) Å	*T* = 100 K
β = 107.995 (3)°	Fragment, colourless
*V* = 1496.56 (13) Å^3^	0.32 × 0.18 × 0.09 mm
*Z* = 4	

### Data collection

**Table:**

Bruker APEXII CCD diffractometer	11218 independent reflections
Radiation source: fine-focus sealed tube	9196 reflections with *I* > 2σ(*I*)
Graphite monochromator	*R*_int_ = 0.054
ω and φ scans	θ_max_ = 43.3°, θ_min_ = 1.9°
Absorption correction: multi-scan (*SADABS*; Bruker, 2013)	*h* = −22→22
*T*_min_ = 0.642, *T*_max_ = 0.749	*k* = −20→20
213856 measured reflections	*l* = −24→24

### Refinement

**Table:**

Refinement on *F*^2^	Primary atom site location: structure-invariant direct methods
Least-squares matrix: full	Secondary atom site location: difference Fourier map
*R*[*F*^2^ > 2σ(*F*^2^)] = 0.021	Hydrogen site location: inferred from neighbouring sites
*wR*(*F*^2^) = 0.046	H-atom parameters constrained
*S* = 1.05	*w* = 1/[σ^2^(*F*_o_^2^) + (0.017*P*)^2^ + 0.2899*P*] where *P* = (*F*_o_^2^ + 2*F*_c_^2^)/3
11218 reflections	(Δ/σ)_max_ = 0.006
215 parameters	Δρ_max_ = 0.48 e Å^−^^3^
20 restraints	Δρ_min_ = −0.52 e Å^−^^3^

### Special details

**Table:**

Geometry. All e.s.d.'s (except the e.s.d. in the dihedral angle between two l.s. planes) are estimated using the full covariance matrix. The cell e.s.d.'s are taken into account individually in the estimation of e.s.d.'s in distances, angles and torsion angles; correlations between e.s.d.'s in cell parameters are only used when they are defined by crystal symmetry. An approximate (isotropic) treatment of cell e.s.d.'s is used for estimating e.s.d.'s involving l.s. planes.
Refinement. Refinement of *F*^2^ against ALL reflections. The weighted *R*-factor *wR* and goodness of fit *S* are based on *F*^2^, conventional *R*-factors *R* are based on *F*, with *F* set to zero for negative *F*^2^. The threshold expression of *F*^2^ > σ(*F*^2^) is used only for calculating *R*-factors(gt) *etc*. and is not relevant to the choice of reflections for refinement. *R*-factors based on *F*^2^ are statistically about twice as large as those based on *F*, and *R*- factors based on ALL data will be even larger.

### Fractional atomic coordinates and isotropic or equivalent isotropic displacement parameters (Å^2^)

**Table:**

	*x*	*y*	*z*	*U*_iso_*/*U*_eq_	
Se1	0.752121 (5)	0.139467 (5)	0.740658 (4)	0.00723 (1)	
Na1	0.74307 (2)	−0.24486 (3)	0.97851 (2)	0.01112 (5)	
Na2	0.75630 (2)	−0.11228 (3)	0.23666 (2)	0.01081 (4)	
O1	0.86910 (4)	0.19770 (5)	0.83551 (4)	0.01211 (7)	
O21	0.73195 (4)	0.21974 (4)	0.62765 (4)	0.01245 (8)	
O31	0.63136 (4)	0.15034 (5)	0.78083 (4)	0.01257 (7)	
O41	0.77699 (5)	−0.00971 (4)	0.71826 (4)	0.01330 (8)	
OW5	0.85363 (4)	−0.21683 (5)	0.85304 (4)	0.01236 (7)	
OW6	0.64473 (4)	−0.27987 (5)	0.11583 (4)	0.01269 (8)	
OW7	0.87675 (5)	0.03985 (5)	0.36196 (4)	0.01443 (8)	
OW8	0.87052 (5)	−0.42968 (5)	0.05311 (4)	0.01472 (8)	
OW9	0.88112 (4)	−0.10907 (5)	0.11623 (4)	0.01306 (8)	
OW10	0.61813 (4)	−0.39325 (5)	0.84832 (4)	0.01310 (8)	
OW11	0.61541 (5)	−0.06034 (5)	0.91589 (4)	0.01584 (9)	
OW12	0.63321 (5)	0.04187 (5)	0.11747 (4)	0.01397 (8)	
OW13	0.09783 (5)	−0.14961 (5)	0.94520 (4)	0.01473 (8)	
OW14	0.39997 (5)	−0.34873 (5)	0.08502 (4)	0.01467 (8)	
H5A	0.8345 (13)	−0.1472 (6)	0.8240 (11)	0.0412 (9)*	
H5B	0.9267 (3)	−0.2110 (14)	0.8845 (10)	0.0412 (9)*	
H6A	0.5712 (2)	−0.2905 (14)	0.1003 (11)	0.0412 (9)*	
H6B	0.6722 (13)	−0.3481 (7)	0.1436 (11)	0.0412 (9)*	
H7A	0.8618 (13)	0.1162 (3)	0.3535 (12)	0.0412 (9)*	
H7B	0.8709 (12)	0.0169 (13)	0.4206 (5)	0.0412 (9)*	
H8A	0.8415 (11)	−0.4602 (12)	0.0978 (8)	0.0412 (9)*	
H8B	0.9440 (2)	−0.4401 (13)	0.0788 (10)	0.0412 (9)*	
H9A	0.9508 (4)	−0.1364 (12)	0.1394 (11)	0.0412 (9)*	
H9B	0.8892 (13)	−0.0359 (5)	0.0973 (11)	0.0412 (9)*	
H10A	0.6143 (13)	−0.4669 (4)	0.8683 (11)	0.0412 (9)*	
H10B	0.5473 (4)	−0.3767 (12)	0.8146 (10)	0.0412 (9)*	
H11A	0.6205 (12)	−0.0062 (10)	0.8719 (8)	0.0412 (9)*	
H11B	0.6254 (12)	−0.0213 (11)	0.9728 (6)	0.0412 (9)*	
H12A	0.6618 (12)	0.1138 (5)	0.1205 (12)	0.0412 (9)*	
H12B	0.5615 (3)	0.0506 (13)	0.1131 (11)	0.0412 (9)*	
H13A	0.1146 (13)	−0.1610 (13)	1.0108 (2)	0.0412 (9)*	
H13B	0.1423 (10)	−0.1952 (11)	0.9226 (10)	0.0412 (9)*	
H14A	0.3586 (11)	−0.3337 (13)	0.0224 (4)	0.0412 (9)*	
H14B	0.3809 (12)	−0.2944 (10)	0.1223 (9)	0.0412 (9)*	

### Atomic displacement parameters (Å^2^)

**Table:**

	*U*^11^	*U*^22^	*U*^33^	*U*^12^	*U*^13^	*U*^23^
Se1	0.00796 (2)	0.00652 (2)	0.00716 (2)	0.00002 (2)	0.00228 (1)	0.00003 (2)
Na1	0.01190 (11)	0.01128 (11)	0.01052 (11)	−0.00036 (9)	0.00396 (9)	−0.00045 (8)
Na2	0.01176 (11)	0.01011 (10)	0.01050 (10)	0.00016 (9)	0.00333 (8)	0.00013 (8)
O1	0.00993 (17)	0.01300 (18)	0.01144 (17)	−0.00124 (14)	0.00041 (14)	−0.00188 (14)
O21	0.0162 (2)	0.01156 (18)	0.00963 (17)	0.00085 (15)	0.00404 (15)	0.00315 (14)
O31	0.00980 (17)	0.0156 (2)	0.01373 (18)	−0.00012 (15)	0.00571 (14)	−0.00071 (15)
O41	0.0190 (2)	0.00658 (16)	0.01498 (19)	0.00140 (15)	0.00617 (16)	−0.00054 (14)
OW5	0.01162 (18)	0.01215 (18)	0.01230 (18)	−0.00102 (15)	0.00222 (14)	0.00144 (14)
OW6	0.01137 (18)	0.01229 (19)	0.01431 (19)	−0.00005 (15)	0.00381 (15)	0.00107 (14)
OW7	0.0160 (2)	0.01212 (19)	0.01404 (19)	−0.00039 (16)	0.00291 (16)	0.00042 (15)
OW8	0.01307 (19)	0.0172 (2)	0.0149 (2)	0.00141 (16)	0.00583 (16)	0.00232 (16)
OW9	0.01073 (18)	0.01301 (18)	0.0154 (2)	0.00022 (14)	0.00393 (15)	0.00012 (15)
OW10	0.01081 (18)	0.01338 (18)	0.01408 (19)	−0.00065 (15)	0.00237 (15)	0.00038 (15)
OW11	0.0166 (2)	0.0150 (2)	0.0176 (2)	0.00205 (17)	0.00788 (17)	0.00367 (16)
OW12	0.01201 (19)	0.01201 (19)	0.0171 (2)	−0.00065 (15)	0.00339 (16)	0.00122 (15)
OW13	0.01312 (19)	0.0176 (2)	0.01342 (19)	0.00137 (16)	0.00410 (15)	0.00051 (16)
OW14	0.0148 (2)	0.0152 (2)	0.01333 (19)	0.00127 (16)	0.00335 (15)	−0.00077 (15)

### Geometric parameters (Å, º)

**Table:**

Na1—OW5	2.3776 (6)	Na2—OW7	2.3935 (6)
Na1—OW6^i^	2.4181 (6)	Na2—OW9	2.4325 (6)
Na1—OW11	2.4184 (6)	Na2—OW6	2.4415 (6)
Na1—OW10	2.4194 (6)	Na2—OW10^ii^	2.4667 (6)
Na1—OW8^i^	2.4473 (6)	Se1—O41	1.6335 (5)
Na1—OW9^i^	2.4507 (6)	Se1—O31	1.6394 (5)
Na2—OW12	2.3814 (6)	Se1—O1	1.6398 (5)
Na2—OW5^ii^	2.3891 (6)	Se1—O21	1.6421 (5)
			
O41—Se1—O31	109.72 (2)	Na1—OW5—H5A	107.3 (10)
O41—Se1—O1	109.88 (3)	Na2^iii^—OW5—H5A	111.8 (10)
O31—Se1—O1	108.89 (2)	Na1—OW5—H5B	111.1 (10)
O41—Se1—O21	108.50 (2)	Na2^iii^—OW5—H5B	125.7 (10)
O31—Se1—O21	110.31 (2)	H5A—OW5—H5B	104.6 (14)
O1—Se1—O21	109.53 (2)	Na1^iv^—OW6—Na2	94.959 (19)
OW5—Na1—OW6^i^	175.60 (2)	Na1^iv^—OW6—H6A	122.0 (10)
OW5—Na1—OW11	94.230 (19)	Na2—OW6—H6A	124.0 (10)
OW6^i^—Na1—OW11	89.467 (19)	Na1^iv^—OW6—H6B	104.4 (10)
OW5—Na1—OW10	86.270 (19)	Na2—OW6—H6B	106.9 (10)
OW6^i^—Na1—OW10	95.70 (2)	H6A—OW6—H6B	102.8 (13)
OW11—Na1—OW10	96.28 (2)	Na2—OW7—H7A	120.4 (10)
OW5—Na1—OW8^i^	88.961 (19)	Na2—OW7—H7B	103.6 (10)
OW6^i^—Na1—OW8^i^	87.276 (19)	H7A—OW7—H7B	109.7 (14)
OW11—Na1—OW8^i^	176.39 (2)	Na1^iv^—OW8—H8A	105.1 (10)
OW10—Na1—OW8^i^	85.59 (2)	Na1^iv^—OW8—H8B	133.6 (10)
OW5—Na1—OW9^i^	93.341 (19)	H8A—OW8—H8B	105.2 (13)
OW6^i^—Na1—OW9^i^	84.368 (19)	Na2—OW9—Na1^iv^	94.36 (2)
OW11—Na1—OW9^i^	88.42 (2)	Na2—OW9—H9A	118.2 (10)
OW10—Na1—OW9^i^	175.30 (2)	Na1^iv^—OW9—H9A	114.2 (10)
OW8^i^—Na1—OW9^i^	89.72 (2)	Na2—OW9—H9B	110.5 (10)
OW12—Na2—OW5^ii^	171.87 (2)	Na1^iv^—OW9—H9B	115.8 (10)
OW12—Na2—OW7	95.37 (2)	H9A—OW9—H9B	104.3 (13)
OW5^ii^—Na2—OW7	90.57 (2)	Na1—OW10—Na2^iii^	92.153 (19)
OW12—Na2—OW9	85.96 (2)	Na1—OW10—H10A	117.7 (10)
OW5^ii^—Na2—OW9	99.063 (19)	Na2^iii^—OW10—H10A	108.3 (10)
OW7—Na2—OW9	95.10 (2)	Na1—OW10—H10B	121.3 (10)
OW12—Na2—OW6	88.92 (2)	Na2^iii^—OW10—H10B	113.9 (10)
OW5^ii^—Na2—OW6	85.252 (19)	H10A—OW10—H10B	103.0 (13)
OW7—Na2—OW6	175.61 (2)	Na1—OW11—H11A	128.3 (10)
OW9—Na2—OW6	84.260 (19)	Na1—OW11—H11B	101.3 (10)
OW12—Na2—OW10^ii^	89.884 (19)	H11A—OW11—H11B	105.1 (13)
OW5^ii^—Na2—OW10^ii^	84.965 (19)	Na2—OW12—H12A	116.2 (10)
OW7—Na2—OW10^ii^	86.209 (19)	Na2—OW12—H12B	120.7 (10)
OW9—Na2—OW10^ii^	175.74 (2)	H12A—OW12—H12B	106.6 (13)
OW6—Na2—OW10^ii^	94.743 (19)	H13A—OW13—H13B	108.1 (13)
Na1—OW5—Na2^iii^	95.178 (19)	H14A—OW14—H14B	105.5 (13)

Symmetry codes: (i) *x*, *y*, *z*+1; (ii) *x*, −*y*−1/2, *z*−1/2; (iii) *x*, −*y*−1/2, *z*+1/2; (iv) *x*, *y*, *z*−1.

### Hydrogen-bond geometry (Å, º)

**Table:**

*D*—H···*A*	*D*—H	H···*A*	*D*···*A*	*D*—H···*A*
O*W*5—H5*A*···O41	0.82 (1)	1.96 (1)	2.7570 (7)	164 (1)
O*W*5—H5*B*···O*W*13^v^	0.82 (1)	2.00 (1)	2.7980 (7)	165 (1)
O*W*6—H6*A*···O*W*14	0.82 (1)	2.02 (1)	2.8301 (7)	168 (1)
O*W*6—H6*B*···O41^ii^	0.82 (1)	1.98 (1)	2.7791 (7)	166 (2)
O*W*7—H7*A*···O1^vi^	0.82 (1)	1.97 (1)	2.7727 (7)	166 (1)
O*W*7—H7*B*···O*W*8^iii^	0.82 (1)	1.95 (1)	2.7542 (7)	168 (1)
O*W*8—H8*A*···O41^ii^	0.82 (1)	1.95 (1)	2.7544 (7)	166 (1)
O*W*8—H8*B*···O*W*7^vii^	0.82 (1)	1.99 (1)	2.8076 (7)	178 (1)
O*W*9—H9*A*···O1^viii^	0.82 (1)	2.11 (1)	2.9152 (7)	168 (1)
O*W*9—H9*B*···O*W*13^ix^	0.82 (1)	2.04 (1)	2.8596 (7)	177 (1)
O*W*10—H10*A*···O*W*14^x^	0.82 (1)	2.05 (1)	2.8686 (7)	178 (2)
O*W*10—H10*B*···O31^xi^	0.82 (1)	2.08 (1)	2.8920 (7)	174 (1)
O*W*11—H11*A*···O31	0.82 (1)	2.05 (1)	2.8604 (7)	171 (1)
O*W*11—H11*B*···O*W*12^i^	0.82 (1)	1.96 (1)	2.7716 (8)	168 (1)
O*W*12—H12*A*···O21^vi^	0.82 (1)	1.92 (1)	2.7359 (7)	179 (1)
O*W*12—H12*B*···O*W*11^ix^	0.82 (1)	1.97 (1)	2.7818 (7)	173 (1)
O*W*13—H13*A*···O1^xii^	0.82 (1)	1.98 (1)	2.7932 (7)	172 (1)
O*W*13—H13*B*···O21^xi^	0.82 (1)	1.98 (1)	2.7931 (7)	170 (1)
O*W*14—H14*A*···O21^xiii^	0.82 (1)	1.98 (1)	2.8002 (7)	174 (1)
O*W*14—H14*B*···O31^ix^	0.82 (1)	2.00 (1)	2.8061 (7)	169 (1)

Symmetry codes: (i) *x*, *y*, *z*+1; (ii) *x*, −*y*−1/2, *z*−1/2; (iii) *x*, −*y*−1/2, *z*+1/2; (v) *x*+1, *y*, *z*; (vi) *x*, −*y*+1/2, *z*−1/2; (vii) −*x*+2, *y*−1/2, −*z*+1/2; (viii) −*x*+2, −*y*, −*z*+1; (ix) −*x*+1, −*y*, −*z*+1; (x) −*x*+1, −*y*−1, −*z*+1; (xi) −*x*+1, *y*−1/2, −*z*+3/2; (xii) −*x*+1, −*y*, −*z*+2; (xiii) −*x*+1, *y*−1/2, −*z*+1/2.
